# Screening Model for Estimating Undiagnosed Diabetes among People with a Family History of Diabetes Mellitus: A KNHANES-Based Study

**DOI:** 10.3390/ijerph17238903

**Published:** 2020-11-30

**Authors:** Kwang Sun Ryu, Ha Ye Jin Kang, Sang Won Lee, Hyun Woo Park, Na Young You, Jae Ho Kim, Yul Hwangbo, Kui Son Choi, Hyo Soung Cha

**Affiliations:** 1 Cancer Big Data Center, National Cancer Center, Goyang-si 10408, Gyeonggi-do, Korea; niceplay13@ncc.re.kr (K.S.R.); khyj0302@ncc.re.kr (H.Y.J.K.); 74915@ncc.re.kr (S.W.L.); nayoung0715@ncc.re.kr (N.Y.Y.); zeoqim@ncc.re.kr (J.H.K.); kschoi@ncc.re.kr (K.S.C.); 2 Healthcare AI Team, National Cancer Center, Goyang-si 10408, Gyeonggi-do, Korea; hwpark@ncc.re.kr (H.W.P.); yulhwangbo@ncc.re.kr (Y.H.); 3 Division of Endocrinology, Department of Internal Medicine, National Cancer Center, Goyang-si 10408, Gyeonggi-do, Korea; 4 Graduate School of Cancer Science and Policy, National Cancer Center, Goyang-si 10408, Gyeonggi-do, Korea

**Keywords:** undiagnosed diabetes mellitus, family history of diabetes, screening model

## Abstract

A screening model for estimating undiagnosed diabetes mellitus (UDM) is important for early medical care. There is minimal research and a serious lack of screening models for people with a family history of diabetes (FHD), especially one which incorporates gender characteristics. Therefore, the primary objective of our study was to develop a screening model for estimating UDM among people with FHD and enable its validation. We used data from the Korean National Health and Nutrition Examination Survey (KNHANES). KNAHNES (2010–2016) was used as a developmental cohort (n = 5939) and was then evaluated in a validation cohort (n = 1047) KNHANES (2017). We developed the screening model for UDM in male (SMM), female (SMF), and male and female combined (SMP) with FHD using backward stepwise logistic regression analysis. The SMM and SMF showed an appropriate performance (area under curve (AUC) = 76.2% and 77.9%) compared with SMP (AUC = 72.9%) in the validation cohort. Consequently, simple screening models were developed and validated, for the estimation of UDM among patients in the FHD group, which is expected to reduce the burden on the national health care system.

## 1. Introduction

Diabetes mellitus affected 422 million people worldwide in 2014, and this number continues to increase [1]. Globally, the proportion of patients with undiagnosed diabetes mellitus (UDM) varies from 30 to 80% [2], with most cases being asymptomatic [3,4]. Early diagnosis allows for optimized treatment of patients with type 2 diabetes mellitus, which helps to achieve good outcomes among individuals with a long asymptomatic disease phase [5,6]. Although the oral glucose tolerance test, fasting plasma glucose level, and hemoglobin A1C level are established biochemical indicators in people with diabetes mellitus [7], it is insufficient to stratify a large population in developing countries [8]. Population-wide screening models for UDM allow for the estimation of individuals at a high risk of developing diabetes without requiring invasive laboratory tests [7].

With these backgrounds, considerable screening models have been previously proposed for various countries and ethnic groups [8,9,10,11,12,13,14,15,16,17]: Glumer et al. developed a simple self-administered questionnaire, identifying individuals with undiagnosed diabetes [8]. Lee et al. developed and validated a self-assessment score for undiagnosed diabetes in the Korean population, based on The Korea National Health and Nutrition Examination Survey (KNHANES) [11]. Katulanda et al. developed a non-invasive screening tool that can classify 80% of undiagnosed diabetes mellitus by selecting 40% of Sri Lankan adults [12]. Heikes et al. developed a simple tool for identifying undiagnosed diabetes mellitus and pre-diabetes mellitus [13]. Zhou et al. developed a screening model for self-assessment of diabetes in a rural Chinese population [8]. Aekplakorn et al. proposed an estimation model for people at a high risk for diabetes mellitus in Thailand [14]. Nanri et al. proposed a prediction model for three-year incidence of type 2 diabetes mellitus in the Japanese population [15]. Gao et al. developed and validated a screening tool for diabetes in Chinese adults [16]. Baan et al. proposed a prediction model to identify individuals who had an increased risk of undiagnosed diabetes [17]. With these backgrounds, previous screening models showed that age and family history of diabetes (FHD) must be included as common variables and generally included age, sex, BMI, family history of diabetes as important variables.

Yang et al. and the authors of this manuscript independently showed that family history of diabetes mellitus (FHD) is significantly correlated with UDM compared to non-FHD [18]. Therefore, we surmised that a practical and meaningful screening model for UDM should be introduced into public health care to reduce UDM in potential high-risk populations. Even though a considerable number of screening models have been developed and are in use, there is a lack of screening models for people with FHD. There has also been minimal research regarding the development of UDM screening models which also incorporate gender characteristics. Consequently, the objective of our study was to develop a practical screening model to estimate the high risk of UDM in people with FHD.

## 2. Materials and Methods

**Figure 1:**
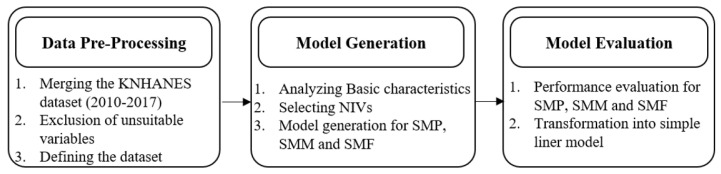
Research framework for the development of a family history of diabetes (FHD) screening model.

The development of UDM screening model for population (SMP) comprising both males and females, male (SMM) population and female (SMF) population in FHD group consists of three steps, as shown in Figure 1. In the first step, KNAHANES datasets collected from 2010 to 2017 were consistently combined. If the scale measurement of a variable was not changed during the study period, it was included in the present study. The combined dataset was pre-processed to obtain a reliable experimental dataset. In the second step, basic characteristics were analyzed comparatively for FHD and non-FHD groups, for male and female populations. Non-invasive variables (NIVs) were selected based on bivariate analysis for each the male, female, and male and female combined populations. These NIVs were used to develop the SMP, SMM, and SMF. These models were evaluated with various measures based on validation datasets in order to generate efficient screening models for people with FHD. Finally, these models were transformed to assign a simple linear risk score to yield a simplified estimate of UDM in people with FHD.

### 2.1. Study Design

All the participants in this survey signed an informed consent form. KNHANES data have been approved by the institutional review board (IRB) of the Korea Center for Disease Control. Approval numbers of 2010–2015 KNHANES are 2010-02CON-21-C, 2011-02CON-06-C, 2012-01EXP-01-2C, 2013-07CON-03-4C, 2013-12EXP-03-5C, and 2015-01-02-6C, respectively. KNHANES VII (2016–2017) was performed without approbation of IRB because this survey is the direct public welfare based on Bioethics law in republic of Korea. Additionally, personal identifiable Information such as name was masked and replaced with unique personal identification numbers. KNHANES data is openly published on website (https://knhanes.cdc.go.kr) [19].

**Figure 2:**
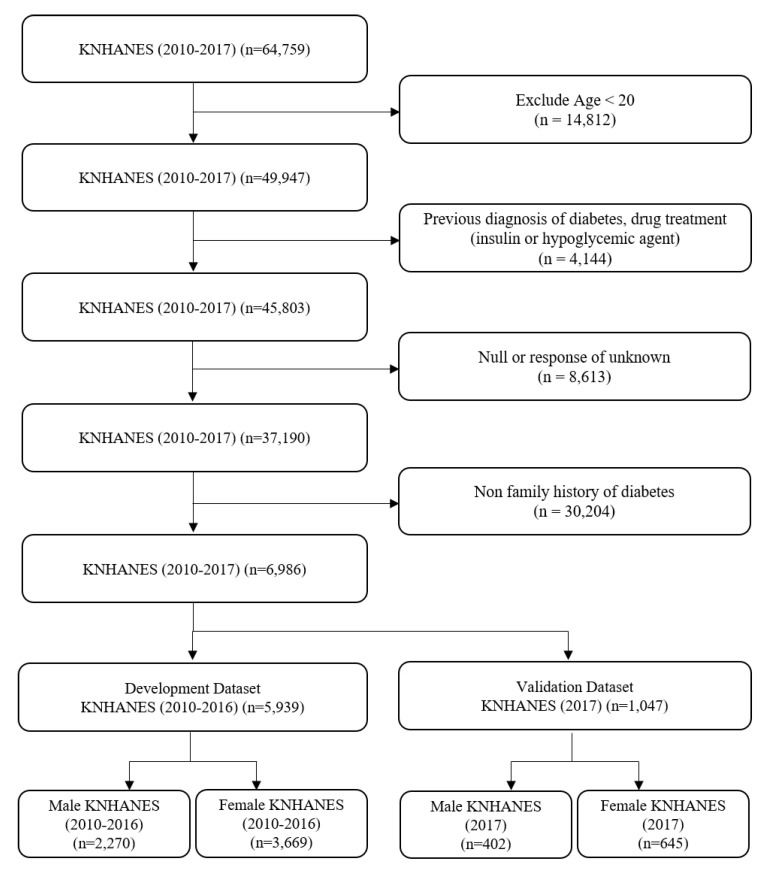
Flow chart for selecting the study population.

KNHANES is a national surveillance system that assesses the health and nutrition status of Koreans, as stipulated in the National Health Promotion Act. KNHANES database includes information on individuals’ health-related behaviors, quality of life, healthcare utilization, anthropometric measures, bio-chemical and clinical profiles [15]. Without duplications, there were 64,759 subjects in the KNHANES 2010–2017 dataset. Those with an age <20 years (n = 14,812), previous diagnosis of diabetes or drug treatments (insulin or hypoglycemic agent) (n = 4144), null or response of unknown (n = 8613), or non-FHD (n = 30,204) were excluded. Study subjects were divided into a development dataset (n = 5939, KNHANES 2010–2016) and validation dataset (n = 1047, KNHANES 2017). Both the development dataset and validation dataset were separated into a male (n = 2270 and 402) and female (n = 3669 and 645) subjects, respectively. These processes are shown in Figure 2.

### 2.2. Definition of Terminology

The UDM was defined as people having fasting plasma glucose (FPG) levels ≥ 126 mg/dL (6.993 mmol/L), having received no previous diagnosis of diabetes mellitus from healthcare professionals and receiving no insulin or oral anti-diabetic agents [11]. Impaired fasting glucose (IFG) was defined as an FPG of 100–125 mg/dL (5.55–6.9375 mmol/L) with above-constraint satisfaction. Individuals were categorized as having FHD including parents or siblings were diagnosed with diabetes mellitus [2]. Body mass index (BMI) was categorized into underweight (BMI < 18.5 kg/m^2^), normal weight (18.5–24.9 kg/m^2^), overweight (25–29.9 kg/m^2^), and obese (BMI ≥ 30 kg/m^2^) [20]. Abdominal obesity was classified as abnormal among males whose waist circumference (WC) was more than 90 cm and among females whose WC was more than 85 cm [20]. Drinking was calculated based on the number of units consumed per week (more than four shots on 5 days per week). Walking indicated a walking workout on more than 6 days per week. Weight training was defined as participating in weight training if for more than a day per week. Stress and depression were incorporated as factors through questionnaires, where participants answered on the levels of stress they felt in their daily lives and the levels of sadness and desperation that interfered with their daily lives for more than two weeks in a row, respectively [19]

### 2.3. Statistical Analyses

Descriptive statistics were used to report participant characteristics. Between-group comparisons were performed with *t*-tests and Chi-square tests; continuous and categorical variables were expressed as mean ± standard deviation (SD) and percentages (%). In the experimental development analyses of the SMP, SMM and SMF, we discretized continuous variables into nominal variables in order to generate scores in the individual populations. Age (Q1: 37, Q2: 45, Q3: 55) and WC (Q1: 74.2, Q2: 80.9, Q3: 87.8) in male and female combined were discretized in accordance with the discretization criterion that was a quartile approach, and BMI was discretized by WHO criteria. In male and female populations, the ages of male (Q1: 37, Q2: 44, Q3: 55) and female (Q1: 37, Q2: 46, Q3: 56) participants were discretized by quartiles based on each distribution, and BMI and WC were discretized by WHO criteria. A univariate logistic regression approach analyzed all potential univariate correlations (*p* < 0.05) in order to generate the multivariate logistic regression model by backward stepwise logistic regression that includes prognosis predictors. We defined scores by rounding down odds ratios (ORs) to first decimal places in the final screening model for estimation of UDM. For example, OR 1.43 was rounded to 1.4 and OR 2.18 was rounded to 2.1. These models were evaluated for sensitivity, specificity, positive predictive value (PPV), negative predictive value (NPV), positive likelihood ratio (PIR), negative likelihood ratio (NIR), Youden index (Youden), and acre under curve (AUC) [12]. These analyses were performed using R software version 3.5.2 (R Foundation for Statistical Computing, Vienna., Austria; http://www.r-project.org/)

## 3. Results

### 3.1. Comparison of Basic Characteristics between Non-FHD Group and FHD Group

The FHD group showed higher body mass index, weight, height, waist circumference, DBP, CHOL, TG, LDL, fasting glucose levels, proportion of females and levels of stress in the high-, mid-high-, mid-low- and low-level categories than the non-FHD group. However, the FHD group was younger, were less likely to have had a previous smoking habit, walked less, had lower incidences of depression, SBP, BUN, Creatinine levels, and hypertension. However, the ratio of IFG and undiagnosed diabetes were higher in the FHD group (Table 1). In order to adjust variable bias, we used a propensity score matching which was used conditional variable such as age, sex, waist circumference, and body mass index. As a result, FHD group showed significant difference as compared with non-FHD group (Table A1). We used a false discovery rate (FDR), and the result of this experiment was statistically significant (Table A2). FRD has shown same variables in Table 1 which are with *p*-value less than 0.05.

### 3.2. Comparison of Basic Characteristics between Male and Female Participants in the FHD Group

The males in the FHD group had higher values of BMI, weight, height, waist circumference, hospital visit rate, drinking, current smoking, previous smoking, walking, weight training, SBP, DBP, CHOL, TG, HB, HCT, BUN, creatinine, fasting glucose level and hypertension. On the contrary, the males in the FHD were younger than the females, had lower levels of depression, HDL and LDL. Furthermore, the proportion of IFG and undiagnosed diabetes were higher in males in the FHD group as shown in (Table A3).

### 3.3. Screening Model Development and Performance Evaluation

Univariate analysis revealed several potential causal factors in the development of undiagnosed diabetes in each population. The group which were combined male and female participants with FHD included variables such as age in years, male, BMI, WC, hypertension, drinking, current smoking, and previous smoking. The male population was age, BMI, WC, hypertension and drinking. Age, BMI, WC, hypertension, and drinking were shown to be significant variables in the female population (Table 2). In multivariate analysis, age, male, BMI, WC, drinking, and hypertension correlated with UDM in the male and female combined. Age, BMI, WC, drinking, and hypertension were associated significantly with UDM in the male population. Age, BMI, WC, and drinking were significant in the female population (Table 3).

**Figure A1:**
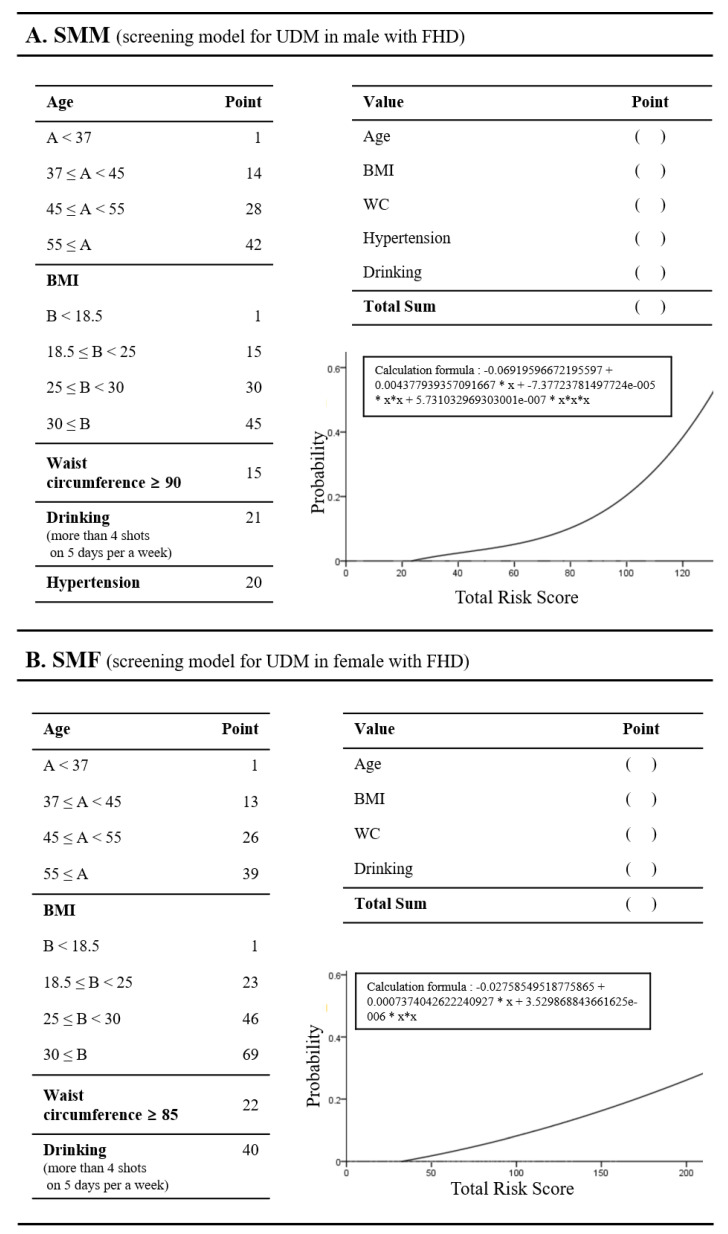
Screening model for estimation of UDM in male and female populations with FHD.

The simplified screening model was created using relevant non-invasive variables based on OR. Continuous variables were divided into four intervals. Values within the first quartile were assigned 1 point and were used as a reference, values in the other quartiles received higher scores, depending on the associated OR. For example, the OR of age in males is 1.43, so people < 37 years of age were assigned 1 point, those 37 ≤ age < 45 were assigned 14 points, those 45 ≤ age < 55 were assigned 28 points, and those 55 ≤ years were assigned 42 points. In the case of categorical variables, the risk score was set to 1 point if the value was no; otherwise, the score increased based on the OR. For instance, hypertension in males had an OR of 2.02, so males with hypertension were assigned 20 points and the other people were assigned 1 point. Furthermore, we defined the nomogram based logistic function to automatically estimate people with UDM, which takes the sum of the scores identified from the score tables in Figure A1. For example, male A is 37 years of age, BMI 30, adnominal waist circumference, drinking, and non-hypertension corresponding to respective risk scores of 14, 45, 15, 21, and with 1 point. The cumulative risk score for people like A is 96 points. The people like A had risk probability of 17.9% for UDM, and the model should recommend people in this category of A have a consultation with an internal medical doctor.

The screening model was verified using the validation dataset (KNHANES 2017) of 1047 people. These data were collected at a different time point to that of the development dataset (KNHANES 2010–2016). We evaluated discriminations with respect to models of UDM, based on the validation dataset using various measures such as sensitivity, specificity, PPV, NPV, PIR, NIR, Youden, and AUC (Table 4). The SMP displayed 57 points with the highest value for Youden of 35.3 with a sensitivity of 87.5%, specificity of 47.8, PPV of 7.5%, NPV of 98.8% and AUC of 72.9. The SMM, with 60 points had the highest value for Youden of 40.4 with a sensitivity of 92.3%, specificity of 48.1%, PPV of 11%, NPV of 98.9%, and AUC of 76.23. The SMF with 82 points showed the highest value for the Youden of 44.6 with a sensitivity of 68.2%, specificity of 76.4%, PPV of 9.3, NPV of 98.6, and AUC of 77.94. Comparing the different model performances, the divided models (SMM and SMF) based on gender showed better performance compared to the integrated model (SMP).

## 4. Discussion

The estimation of people with UDM is important in ensuring positive patient outcomes and preventing complications. Previous studies from various countries and with diverse populations have shown adequate goodness of fit and satisfactory validity of the proposed models for identifying individuals with UDM. [8,10,11,12,13,14,15,16,17]. These studies centered on the estimation of UDM in the general population. In contrast to these studies, our model concentrated on specific smaller populations, namely male and female with FHD. In our experimental results, people with FHD displayed a variety of adverse indicators including higher BMI, weight, waist circumference, CHOL, DBP, TG, LDL, fasting glucose level, higher frequency of stress, and lower frequency of walking compared to people without FHD. Although people with FHD tended to be younger, had a lower frequency of depression, previous smoking, hypertension, lower BUN, and creatinine levels, this group showed significantly higher rates of IFG and UDM, compared to the non-FHD population. Especially, we used a propensity score matching to adjust differences of the basic clinical characteristics and unbalanced number of people in the FHD and non-FHD group. Conditional variables were used age, sex, waist circumference, and body mass index. After adjustment basic characteristic, the FHD group showed significant difference as compared with non-FHD (Table A1). Additionally, multivariate analysis result for non-FHD showed significantly difference as compared with FHD results (Table A4). Furthermore, male population in FHD showed a higher BMI, waist circumference, drinking, current smoking, previous smoking, SBP, DBP, CHOL, HDL, TG, BUN, creatinine, fasting glucose, and hypertension compared to the female population. This group especially showed significantly higher frequencies of IFG and UDM. With these backgrounds, we focused on establishing screening models dedicated to male and female populations with FHD.

In order to reflect the characteristics of male and female populations in FHD, we generated SMM and SMF, respectively. Common estimation factors in each model were age, BMI, waist circumference, and drinking. Age was a major risk factor in the model, as age is strongly associated with an increased risk of diabetes mellitus [2,21,22]. Our model included an estimation factor for aging which was divided into quartiles according to the respective gender distributions. Waist circumference is considered a reliable indicator of future diabetes mellitus risk [23,24,25]. In our experimental model, abnormality in waist circumference was differentially defined between male and female populations, according to WHO criteria [20]. Drinking is associated with disruption of the glucose metabolism, including its effects on the muscle, liver, and adipose tissue, as observed under baseline conditions and following stimulation [26,27,28,29]. Although drinking was significantly higher among the male population compared to the female population, this factor was equally included in the estimation models. BMI is an important variable to estimate UDM in this model [30,31,32,33], we used BMI according to the WHO criteria [20]. Hypertension is an important comorbidity among patients with diabetes [34,35], and has significantly higher incidence among diabetics compared to the general population [36,37,38]. Hypertension occurrence is higher among males than females, regardless of race and ethnicity [39,40,41]. In our experimental model, hypertension was the only differential factor between SMM and SMF in a comparison based on estimation factors. Hypertension is therefore uniquely associated with UDM among the male population with FHD.

Compared to previous studies, the differentiation between male and female populations with FHD makes our screening model unique. Significant differences in experimental results were observed in not only the basic characteristics between male and female populations but also the components of SMM and SMF. Crucially, SMM and SMF showed more appropriate performance compared to SMP. We therefore recommend subdividing the population based on sexes to generate models for the estimation of UDM. The models were evaluated to guarantee reliability based on the validation dataset (KNHANES 2017) being assembled at a different timepoint to the development dataset (KNHANES 2010-2016).

Our study has some limitations. First, FHD and non-FHD groups showed unbalance distribution, which should cause bias result that ignoring people without FHD. Second, this study has been carried out very specific dataset in a country. Third, recently, machine learning algorithms were applied to develop the screening model in various clinical area. Our study focused on statistical model based on logistic regression. In order to overcome these limitations, a randomized, prospective, large volume clinical trial and various approach such as machine learning should be clearly required in future work.

## 5. Conclusions

The proposed screening models included non-invasive variables, which can be used in large populations. This simple and practical tool can be used by patients with limited access to clinical examinations, including blood tests. These screening models showed good predictive performance that was transformed into a simple linear screening score, which can be easily calculated. Future studies should examine larger populations and analyze the impact of comorbidity and chronic disease. Moreover, research on relevant predictive models are needed based on multinational standards.

## Figures and Tables

**Table 1:** Baseline characteristics of people with FHD and without non-FHD.

Variable	Non-FHD (*n* = 30,204)	FHD (*n* = 6986)	*p*-Value
**Demographic characteristics**			
Age (years)	49.9 ± 16.3	46.5 ± 13.0	0.01
Body mass index (kg/m^2^)	23.6 ± 3.4	24.0 ± 3.5	0.01
Weight (kg)	62.7 ± 11.8	64.2 ± 12.3	0.01
Height (m)	1.63 ± 0.1	1.63 ± 0.1	0.01
Waist circumference (m)	0.80 ± 0.1	0.81 ± 0.1	0.01
Female (%)	56.6	61.8	0.01
**Social Factors**			
Hospital visit rate (%)	42.4	82.4	0.69
Stress (%)			0.01
High	4.2	4.7	
Mid-high	21.4	23.8	
Mid-low	58.2	59.7	
Low	16.2	11.8	
Depression (%)	9.5	4.7	0.01
**Lifestyle Factors**			
Drinking (%)	4.5	4.2	0.29
Current Smoking (%)	19.2	20.1	0.08
Previous Smoking (%)	20.7	17.4	0.01
Walking (%)	33.6	31.9	0.01
Weight training	25.1	25.2	0.84
**Clinical Factors**			
Systolic blood pressure (mmHg)	118.6 ± 17.0	116.5 ± 15.7	0.01
Diastolic blood pressure (mmHg)	75.8 ± 10.3	76.2 ± 10.2	0.01
Total cholesterol (mg/dL)	191.4 ± 35.8	194.2 ± 35.7	0.01
High density lipoprotein ST2 (mg/dL)	51.1 ± 12.3	51.2 ± 12.3	0.84
Triglyceride (mg/dL)	128.3 ± 91.9	134.9 ± 100.6	0.01
Low density lipoprotein cholesterol (mg/dL)	114.6 ± 32.3	116.0 ± 32.3	0.01
Hemoglobin (g/dL)	14.0 ± 1.6	14.0 ± 1.6	0.17
Hematocrit (%)	42.2 ± 4.2	42.2 ± 4.2	0.98
Blood urea nitrogen (mg/dL)	14.3 ± 4.4	13.9 ± 4.0	0.01
Creatinine (mg/dL)	0.83 ± 0.2	0.80 ± 0.3	0.01
Fasting glucose (mg/dL)	95.2 ± 14.3	97.7 ± 19.4	0.01
Hypertension (%)	20.3	13.8	0.01
Impaired fasting glucose (%)	23.1	26.1	0.01
Undiagnosed diabetes (%)	2.7	4.5	0.01

Data are expressed as percentage or mean ± SD. FHD, family history of diabetes; Drinking was calculated based on the number of units consumed per week: more than 4 shots on 5 days per week. Walking, walking workout more than 6 days per week; Weight training, participating in weight training more than a day per week; Stress, stress recognition by self; Depression, felt sad or desperate enough to interfere with your daily life for more than two weeks in a row, during the past year.

**Table 2:** Univariate Analysis of people with FHD.

Male and Female Combined			
Variable	B	Odds (95% CI)	*p*-Value
Age	0.35	1.42 (1.27–1.60)	0.01
Male	0.68	1.97 (1.54–2.52)	0.01
Body mass index	0.95	2.59 (2.18–3.07)	0.01
Waist circumference	0.76	2.14 (1.87–2.45)	0.01
Hypertension	1.12	3.07 (2.34–4.02)	0.01
Drinking	1.26	3.53 (2.39–5.21)	0.01
Current Smoking	0.30	1.35 (1.02–1.79)	0.04
Previous Smoking	0.48	1.62 (1.22–2.16)	0.01
Walking	−0.16	0.85 (0.65–1.12)	0.25
Weight training	−0.19	0.83 (0.62–1.11)	0.20
Hospital visit rate	0.06	1.06 (0.75–1.51)	0.73
Stress	−0.02	0.98 (0.82–1.16)	0.80
Depression	−0.12	0.89 (0.55–1.44)	0.64
**Male**			
Age	0.41	1.51 (1.28–1.78)	0.01
Body mass index	0.56	1.75 (1.36–2.25)	0.01
Waist circumference	0.87	2.38 (1.70–3.35)	0.01
Hypertension	1.13	3.08 (2.13–4.46)	0.01
Drinking	0.92	2.51 (1.62–3.89)	0.01
Current Smoking	−0.12	0.89 (0.63–1.25)	0.50
Previous Smoking	0.28	1.32 (0.94–1.86)	0.11
Walking	−0.10	0.90 (0.63–1.29)	0.58
Weight training	−0.28	0.75 (0.52–1.08)	0.13
Hospital visit rate	0.05	0.95 (0.57–1.59)	0.86
Stress	0.01	1.01 (0.79–1.28)	0.96
Depression	0.86	0.42 (0.13–1.35)	0.15
**Female**			
Age	0.37	1.45 (1.22–1.72)	0.01
Body mass index	1.26	3.53 (2.77–4.50)	0.01
Waist Circumference	1.80	6.04 (4.15–8.79)	0.01
Hypertension	1.08	2.94 (1.97–4.40)	0.01
Drinking	1.54	4.65 (1.78–12.16)	0.01
Current Smoking	0.14	1.15 (0.55–2.38)	0.72
Previous Smoking	−0.23	0.80 (0.35–1.84)	0.60
Walking	−0.32	0.73 (0.48–1.11)	0.73
Weight training	−0.46	0.63 (0.37–1.07)	0.09
Hospital visit rate	0.04	1.04 (0.64–1.69)	0.88
Stress	0.05	1.05 (0.82–1.36)	0.70
Depression	0.32	1.38 (0.81–2.37)	0.24

B indicates beta coefficient; CI, confidence interval.

**Table 3:** Multivariate Analysis of People with FHD.

Male and Female Combined			
Variable	B	Odds (95% CI)	*p*-Value
Age	0.25	1.28 (1.12–1.46)	0.01
Male	0.30	1.35 (1.02–1.79)	0.03
Body mass index	0.55	1.74 (1.36–2.23)	0.01
Waist circumference	0.38	1.46 (1.21–1.76)	0.01
Drinking	0.86	2.37 (1.56–3.60)	0.01
Hypertension	0.56	1.76 (1.30–2.37)	0.01
**Male**			
Age	0.36	1.43 (1.20–1.71)	0.01
Body mass index	0.40	1.50 (1.06–2.12)	0.02
Waist circumference	0.45	1.58 (1.01–2.46)	0.05
Drinking	0.78	2.18 (1.38–3.42)	0.01
Hypertension	0.70	2.02 (1.36–3.01)	0.01
**Female**			
Age	0.26	1.30 (1.09–1.55)	0.01
Body mass index	0.86	2.37 (1.67–3.35)	0.01
Waist circumference	0.83	2.29 (1.36–3.86)	0.01
Drinking	1.40	4.07 (1.44–11.55)	0.01

B indicates beta coefficient; CI, confidence interval.

**Table 4:** Performance evaluation of the models of undiagnosed diabetes mellitus (UDM).

**SMP**								
**Point**	**Sensitivity**	**Specificity**	**PPV**	**NPV**	**PIR**	**NIR**	**Youden**	**AUC**
45	95.8	23.2	5.7	99.1	1.25	0.18	19.0	72.9
57	87.5	47.8	7.5	98.8	1.68	0.26	35.3	72.9
98	50.0	76.2	9.2	96.9	2.10	0.66	26.2	72.9
**SMM**								
46	100	23.9	8.3	100	1.31	0.00	23.9	76.23
60	92.3	48.1	11	98.9	1.78	0.16	40.4	76.23
75	65.4	71.0	13.5	96.7	2.26	0.49	36.4	76.23
**SMF**								
38	100	13.8	3.9	100	1.16	0.00	13.8	77.94
59	86.4	51.0	5.9	99.1	1.76	26.7	37.4	77.94
82	68.2	76.4	9.3	98.6	2.89	41.6	44.6	77.94

PPV, positive predictive value; NPV, negative predictive value; PIR, positive likelihood ratio; NIR, negative likelihood ratio; Youden, Youden index; AUC, acre under curve.

## Data Sharing Statement

The dataset used during the current study are available from the corresponding authors upon reasonable request.

## Appendix A

**Table A1:** Propensity score-matched baseline characteristics of people with (FHD) and without (non-FHD) family history of diabetes.

Variable	Non-FHD (*n* = 6986)	FHD (*n* = 6986)	*p*-Value
**Demographic characteristics**			
Age (years)	46.3 ± 15.3	46.5 ± 13	0.37
Body mass index (kg/m^2^)	23.9 ± 3.53	24 ± 3.5	0.08
Weight (kg)	63.5 ± 12.2	64.2 ± 12.3	0.00
Height (Cm)	163 ± 9.22	163 ± 8.9	0.00
Waist circumference (Cm)	81.1 ± 10.2	81.4 ± 9.9	0.06
Female (%)	62.3	61.8	0.5
**Social Factors**			
Hospital visit rate (%)	84.5	84.7	0.74
Stress (%)			0.00
High	4.3	4.7	
Mid-high	23.0	23.8	
Mid-low	58.9	59.7	
Low	13.8	11.8	
Depression (%)	9.0	8.3	0.14
**Lifestyle Factors**			
Drinking (%)	3.9	4.2	0.39
Current Smoking (%)	18.5	20.1	0.02
Previous Smoking (%)	17.1	17.4	0.59
Walking (%)	33.1	31.9	0.13
Weight training (%)	25.4	25.2	0.85
**Clinical Factors**			
Systolic blood pressure (mmHg)	117 ± 16.3	116 ± 15.7	0.02
Diastolic blood pressure (mmHg)	75.9 ± 10.3	76.2 ± 10.2	0.16
Total cholesterol (mg/dL)	191 ± 36	194 ± 35.6	<0.001
High density lipoprotein ST2 (mg/dL)	51.5 ± 12.3	51.2 ± 12.3	0.08
Triglyceride (mg/dL)	127 ± 93.2	135 ± 101	<0.001
Low density lipoprotein cholesterol (mg/dL)	114 ± 32.3	116 ± 32.3	0.00
Hemoglobin (g/dL)	14 ± 1.58	14 ± 1.6	0.45
Hematocrit (%)	42 ± 4.2	42.2 ± 4.2	0.06
Blood urea nitrogen (mg/dL)	13.8 ± 4.04	13.9 ± 4	0.19
Creatinine (mg/dL)	0.81 ± 0.23	0.81 ± 0.3	0.68
Fasting glucose (mg/dL)	94.9 ± 15.1	97.7 ± 19.4	<0.001
Hypertension (%)	16.8	13.8	<0.001
Impaired fasting glucose (%)	21.6	26.1	<0.001
Undiagnosed diabetes (%)	2.6	4.5	<0.001

Data are expressed as percentage or mean ± SD. FHD, family history of diabetes; Drinking was calculated based on the number of units consumed per week: more than 4 shots on 5 days per week. Walking, walking workout more than 6 days per week; Weight training, participating in weight training more than a day per week; Stress, stress recognition by self; Depression, felt sad or desperate enough to interfere with your daily life for more than two weeks in a row, during the past year.

**Table A2:** Baseline characteristics of people with FHD and without non-FHD based on FDR.

Variable	Non-FHD (*n* = 30,204)	FHD (*n* = 6986)	*p*-Value	Adjusted *p*-Value *
**Demographic characteristics**				
Age (years)	49.9 ± 16.3	46.5 ± 13.0	0.01	0.01
Body mass Index (kg/m^2^)	23.6 ± 3.4	24.0 ± 3.5	0.01	0.01
Weight (kg)	62.7 ± 11.8	64.2 ± 12.3	0.01	0.01
Height (m)	1.63 ± 0.1	1.63 ± 0.1	0.01	0.01
Waist circumference (m)	0.80 ± 0.1	0.81 ± 0.1	0.01	0.01
Female (%)	56.6	61.8	0.01	0.01
**Social Factors**				
Hospital visit rate (%)	42.4	82.4	0.69	0.77
Stress (%)			0.01	0.01
High	4.2	4.7		
Mid-high	21.4	23.8		
Mid-low	58.2	59.7		
Low	16.2	11.8		
Depression (%)	9.5	4.7	0.01	0.01
**Lifestyle Factors**				
Drinking (%)	4.5	4.2	0.29	0.34
Current Smoking (%)	19.2	20.1	0.08	0.1
Previous Smoking (%)	20.7	17.4	0.01	0.01
Walking (%)	33.6	31.9	0.01	0.01
Weight training	25.1	25.2	0.84	0.87
**Clinical Factors**				
Systolic blood pressure (mmHg)	118.6 ± 17.0	116.5 ± 15.7	0.01	0.01
Diastolic blood pressure (mmHg)	75.8 ± 10.3	76.2 ± 10.2	0.01	0.01
Total cholesterol (mg/dL)	191.4 ± 35.8	194.2 ± 35.7	0.01	0.01
High density lipoprotein ST2 (mg/dL)	51.1 ± 12.3	51.2 ± 12.3	0.84	0.87
Triglyceride (mg/dL)	128.3 ± 91.9	134.9 ± 100.6	0.01	0.01
Low density lipoprotein cholesterol (mg/dL)	114.6 ± 32.3	116.0± 32.3	0.01	0.01
Hemoglobin (g/dL)	14.0±1.6	14.0 ± 1.6	0.17	0.21
Hematocrit (%)	42.2 ± 4.2	42.2 ± 4.2	0.98	0.98
Blood urea nitrogen (mg/dL)	14.3 ± 4.4	13.9 ± 4.0	0.01	0.01
Creatinine (mg/dL)	0.83 ± 0.2	0.80 ± 0.3	0.01	0.01
Fasting glucose (mg/dL)	95.2 ± 14.3	97.7 ± 19.4	0.01	0.01
Hypertension (%)	20.3	13.8	0.01	0.01
Impaired fasting glucose (%)	23.1	26.1	0.01	0.01
Undiagnosed diabetes (%)	2.7	4.5	0.01	0.01

Data are expressed as percentage or mean ± SD. FHD, family history of diabetes; Drinking was calculated based on the number of units consumed per week: more than 4 shots on 5 days per week. Walking, walking workout more than 6 days per week; Weight training, participating in weight training more than a day per week; Stress, stress recognition by self; Depression, felt sad or desperate enough to interfere with your daily life for more than two weeks in a row, during the past year.

**Table A3:** Baseline characteristics of male and female participants with family history of diabetes (FHD).

Variable	Male (*n* = 2672)	Female (*n* = 4314)	*p*-Value
**Demographic characteristics**			
Age (years)	46.0 ± 12.9	46.9 ± 13.1	0.01
Body mass index (kg/m^2^)	24.7 ± 3.3	23.6 ± 3.4	0.01
Weight (kg)	72.7 ± 11.8	59.0 ± 9.4	0.01
Height (m)	1.71 ± 0.1	1.58 ± 0.1	0.01
Waist circumference (m)	0.86 ± 0.1	0.79 ± 0.1	0.01
**Social Factors**			
Hospital visit rate	87.3	83.1	0.01
Stress			0.10
High	4.0	5.2	
Mid-high	24.4	23.4	
Mid-low	59.7	59.8	
Low	12.0	11.6	
Depression	5.0	10.3	0.01
**Lifestyle Factors**			
Drinking (%)	9.3	1.1	0.01
Current Smoking (%)	43.3	5.7	0.01
Previous Smoking (%)	35.9	6.1	0.01
Walking (%)	34.5	30.3	0.01
Weight training (%)	35.1	19.1	0.01
**Clinical Factors**			
Systolic blood pressure (mmHg)	119.8 ± 14.1	114.4 ± 16.3	0.01
Diastolic blood pressure (mmHg)	79.9 ± 10.0	73.8 ± 9.6	0.01
Total cholesterol (mg/dL)	195.4 ± 35.1	193.4 ± 36.0	0.03
High density lipoprotein ST2 (mg/dL)	46.6 ± 10.9	54.0 ± 12.2	0.01
Triglyceride (mg/dL)	170.5 ± 125.4	112.9 ± 73.4	0.01
Low density lipoprotein cholesterol (mg/dL)	114.7 ± 33.5	116.9 ± 31.5	0.01
Hemoglobin (g/dL)	15.4 ± 1.1	13.1 ± 1.2	0.01
Hematocrit (%)	45.9 ± 3.1	39.9 ± 3.1	0.01
Blood urea nitrogen (mg/dL)	14.7 ± 3.9	13.3 ± 4.0	0.01
Creatinine (mg/dL)	0.9 ± 0.3	0.7 ± 0.2	0.01
Fasting glucose (mg/dL)	101.0 ± 22.5	95.6 ± 16.8	0.01
Hypertension (%)	15.1	13.0	0.02
Impaired fasting glucose (%)	33.2	21.7	0.01
Undiagnosed diabetes (%)	6.4	3.4	0.01

Data are expressed as percentage or mean ± SD. FHD, family history of diabetes; Drinking was calculated based on the number of units consumed per week: more than 4 shots on 5 days per a week; Walking, walking workout more than 6 days per week; Weight training, participating in weight training more than a day per week; Stress, stress recognition by self; Depression, felt sad or desperate enough to interfere with your daily life for more than two weeks in a row, during the past year.

**Table A4:** Multivariate Analysis of people with Non-FHD.

People			
Variable	B	Odds (95% CI)	*p*-Value
Age	0.21	1.24 (1.08–1.42)	0.00
WC	0.68	1.98 (1.72–2.28)	0.00
Hypertension	0.56	1.75 (1.29–2.38)	0.00
Drinking	0.94	2.55 (1.71–3.82)	0.00

B indicates beta coefficient; CI, confidence interval; BMI, body mass index; WC, waist circumference.
